# The Dental Meetings at Saratoga

**Published:** 1889-09

**Authors:** 


THE DENTAL MEETINGS AT SARATOGA.—Much of importance to the profession of dentistry was done at the meetings of the American Dental Association, The National Association of Dental Faculties, and The National Association of Dental Examining Boards, held at Saratoga, New York, in August, 1889. Dr. Allport gives the following summary of what was accomplished:

"That of the greatest importance is the action in regard to the extending of the course of instruction in dental Colleges. To understand this perfectly one should know something of what has been previously done and what action seems necessary at this time. Within fifty years the practice of dentistry, as far as developed, was simple and embraced but little—the mere mechanical work of pulling teeth, with but little knowledge of the nature or causes of disease; the making of plain sets of artificial teeth. About forty years ago the need of better qualifications had so increased that a dental college was established at Baltimore and the degree of D. D. S. was created. Previous to that time all instruction was given in private offices and the usual course was six months of office instruction. But the college required attendance on two courses of lectures of three months each, in which was comfortably taught all then known of the principals of dentistry.

As knowledge increased and better skill was demanded the courses of instruction were made four months and other colleges were established until now there are over thirty in the United states of quite different courses and lengths of terms. Some of these have two years and terms of four or five months, others six and seven months and some three terms of six months; still others three of nine months. Experience has taught that three years is as little time as the average young man can with the very best instruction qualify himself in to properly practice dentistry. To understand this may be somewhat difficult for the laity, but did they understand the importance of this preparation they would insist that all young graduates they employ should have had it.

Dentistry is a peculiar calling, requiring a variety of talents and qualifications as the mechanism is exceedingly intricate and involves some of the most important principles. Take if you please, making artificial teeth. When practiced with the skill which its importance demands it is a difficult mechanical pursuit. It involves manipulation of gold in many cases as intricate as in any branch of jewelry; of working platina and fusing minerals; of making gum bodies and imitating the natural gum, and the manipulation of rubber and other materials for that purpose. Each requires a different system. Filling teeth simply as a mechanical operation is a most difficult pursuit and great excellence is reached by but few. Then in artificial teeth it requires such a knowledge of of art that when properly done the denture shall be so life-like in color and shape and so conformed to the contour and grace lines of the face that they should conceal the fact that they are artificial.

Now here are two trades and an art combined in the filling of teeth and making of artificial teeth. Added to this a dentist should be as well educated in the fundamental principles of medicine as he should be if he were to treat the various diseases of the eye, ear or any other separate organ of the body. To understand this he must understand general anatomy, the general principles of chemistry physiology, pathology, the nature of therapeutical remedies as well as the general principals of surgery. In fact no man can make an intelligent practitioner in the treatment and care of any disease of the body unless he understands the general principles that underlie the treatment of all other diseases. To meet the demand of all dental colleges have partial courses of medical teachings and some of the schools are in connection with medical colleges where these principles are taught by regular medical professors as fully as in the teaching of medical students, and they are required to pass the same examination.

To meet the demand in regard to teaching in various departments the colleges determined that terms ought to be three years and each term not less than five months. Some extend the terms to three, or five and six or nine months.

At the present meeting of the American Dental Assciation a resolution has been passed that no college should be represented in the association that did not comply with the regulation established by this national college association of dental faculties, which is three years or three terms of not less than five months each.

To enforce this: The public will better realize the importance of this action when it remembers the fact that it takes not less than three years to make a brick mason, shoemaker or carpenter and the proper understanding of dentistry requires the application of the most intricate form of mechanics in at least two trades, good general ideas of art and the important principals of medical science.

No meeting of the association ever held has done as much to promote and secure an intelligent practice of dentistry and benefit the community."

The meeting was really a tripartite affair and at once the meeting of the American Dental Association, of the Association of Dental Faculties and the Association of state dental examiners. No such gathering of dentists has ever been held in Saratoga or anywhere else.

